# Supplementary material for: Acetylation regulates ribonucleotide reductase activity and cancer cell growth
Source: Nat Commun. 2019 Jul 19;10:3213. doi: 10.1038/s41467-019-11214-9 (PMC6642173; doi:10.1038/s41467-019-11214-9)
Supplement: Supplementary file 1 — Supplementary Information [file 41467_2019_11214_MOESM1_ESM.pdf]

Supplementary Information

Acetylation regulates ribonucleotide reductase activity and cancer cell growth

Guo Chen, Yin Luo, Kurt Warncke, Youwei Sun, David S. Yu, Haian Fu, Madhusmita Behera, Suresh S. Ramalingam, Paul W. Doetsch, Duc M. Duong, Michael Lammers, Walter J. Curran, and Xingming Deng

Supplementary Data

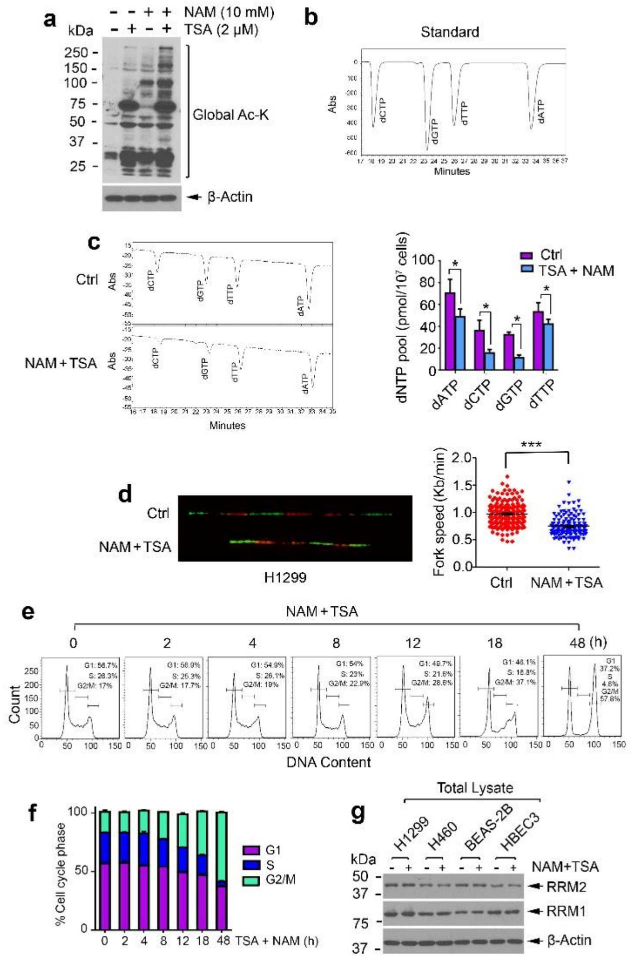

Supplementary Fig. 1 Treatment of cells with deacetylase inhibitors leads to reduction of intracellular dNTPs and retardation of DNA replication fork progression. (**a**) H1299 cells were treated with NAM (10 mM) or TSA (2 µM) alone or in combination for 18h, followed by Western blot using pan-acetyl lysine antibody. (**b**) HPLC separation of a standard mixture of dATP, dCTP, dGTP, and dTTP. (**c**) H1299 cells were treated with a combination of NAM (10 mM) and TSA (2 µM) for 18 h, followed by measurement of intracellular dNTPs by HPLC. The error bars indicate ± s.d. of three separate experiments. *$P < 0.05$, by 2-tailed t test. (**d**) Cells were pulsed-labeled with 100µM CldU for 20 min and 100 µM IdU for another 20 min. The labeled cells were processed for DNA combing. Representative pairs of sister replication forks are shown. Red, CldU. Green, IdU. The DNA replication fork rates were quantified. The error bars indicate ± s.d., n = 150 fibers. ***$P < 0.001$, by 2-tailed t test. (**e**) and (**f**) H1299 cells were treated with combination of NAM (10mM) and TSA (2µM) for a time course up to 48h, followed by analysis of cell cycle by FACS. (**g**) H1299 cells were treated with a combination of NAM (10mM) and TSA (2µM) for 18 h, followed by Western blot using RRM1 or RRM2 antibody, respectively. Source data are provided as a Source Data file.

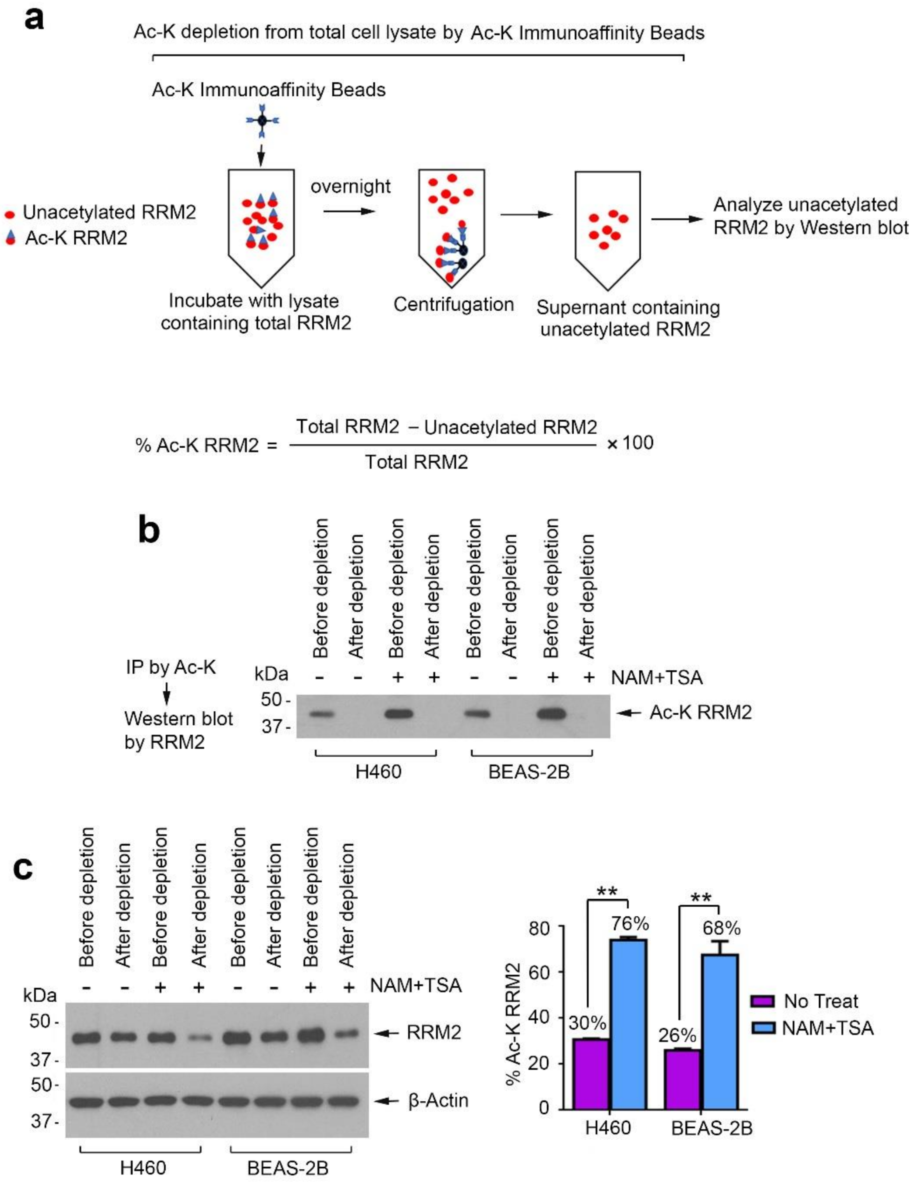

Supplementary Fig. 2 Quantification of Ac-K RRM2 before and after treatment with NAM and TSA. (**a**) Schematic diagram of Ac-K depletion from cell lysate and measurement of % acetylated-RRM2 (Ac-K RRM2). (**b**) Confirm depletion efficiency via measurement of Ac-K RRM2 by IP using Ac-K specific antibody in the lysates before vs. after Ac-K depletion, followed by Western blot analysis of Ac-K RRM2 using anti-RRM2 antibody. (**c**) Measurement of total RRM2 and unacetylated RRM2 in the lysates before and after Ac-K depletion in H460 and BEAS-2B cells with and without NAM/TSA treatment, followed by quantifying the unaceylated RRM2 and total RRM2 on Western blot bands using ImageJ software and calculation using formula: % Ac-K RRM2 = (Total RRM2-Unacetylated RRM2)/Total RRM2 ×100. The error bars indicate ± s.d. of three separate experiments. ***P* < 0.01, by 2-tailed *t* test. Source data are provided as a Source Data file.

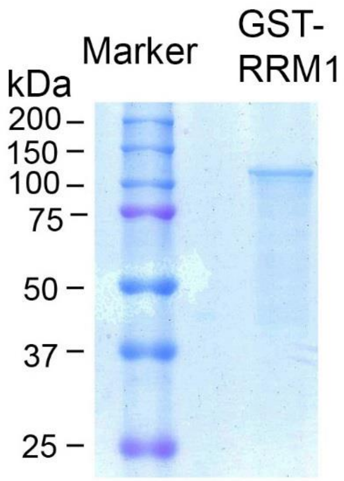

Supplementary Fig. 3 Coomassie blue staining of purified recombinant GST-RRM1 protein from *E.coli.*

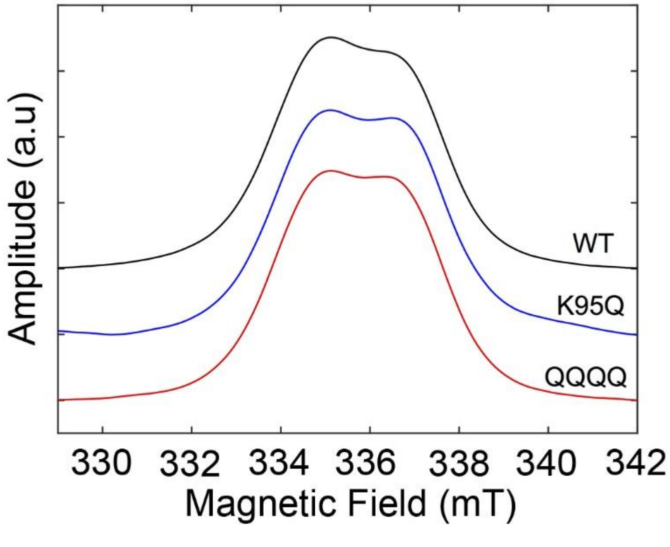

Supplementary Fig. 4 CW-EPR absorption spectra provide the area under the curve, which is proportional to the number of electron spins in the sample.

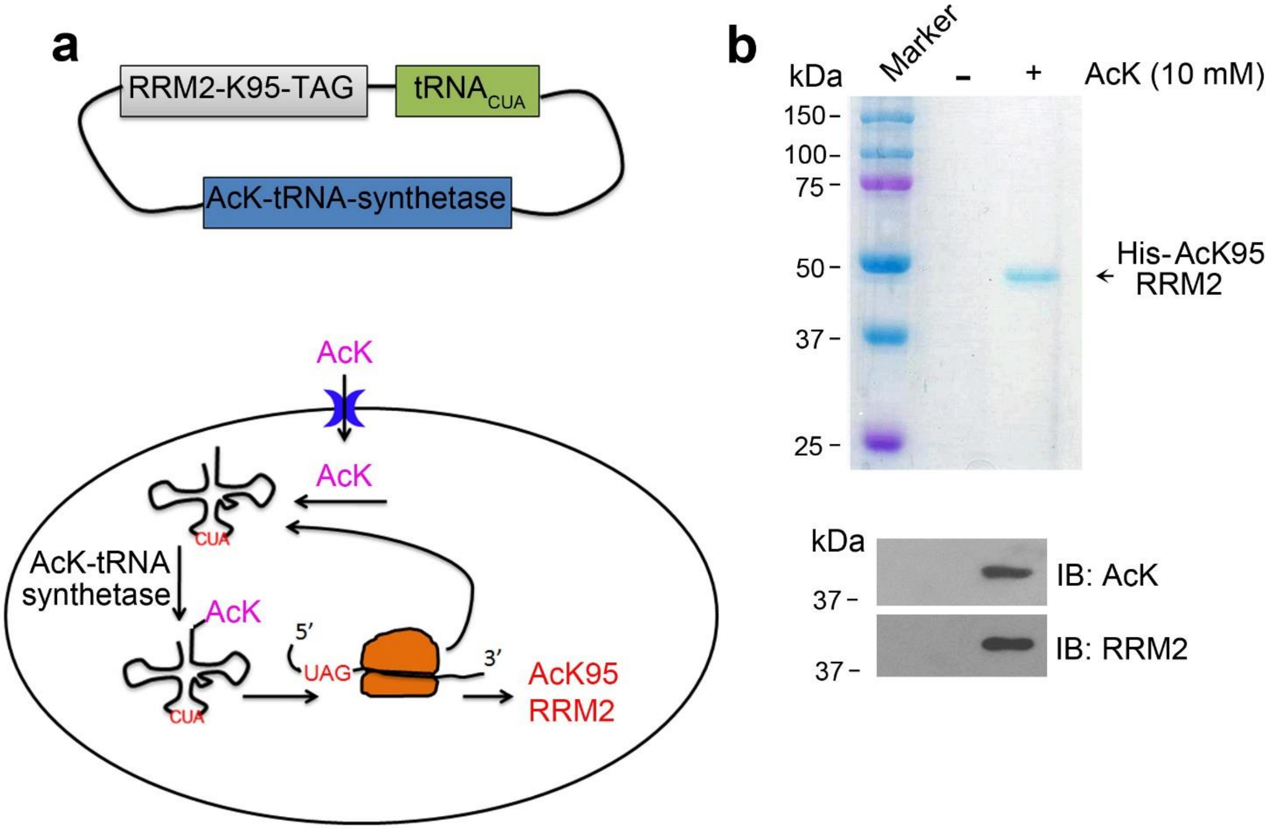

Supplementary Fig. 5 Generation of RRM2 AcK95 protein. (**a**) Diagram of the principle of site-specific incorporation of Nε-acetyl-L-lysine (AcK) into RRM2 using the genetic code expansion concept. The pRSF-Duet1/MbtRNA$_{CUA}$/AcKRS-3 construct contains the coding regions for acetyl-lysyl-tRNA-synthetase (AcKRS3) and the cognate amber suppressor MbtRNA$_{CUA}$ from *Methanosarcina barkeri*. Human RRM2 cDNA was cloned into the pRSF-Duet1-MbtRNA$_{CUA}$/AcKRS-3, followed by mutating RRM2 K95 (AAG) to TAG, which can exclusively be recognized by MbtRNA$_{CUA}$ for site-specific acetyl-lysine incorporation. The resulting acetyl K95 RRM2 (AcK95-RRM2) constructs were transformed into *E.coli* to produce recombinant AcK95 protein with supplement of N$_\varepsilon$-acetyl-L-lysine in LB medium. (**b**) The recombinant RRM2 AcK95 protein was expressed and purified after supplementation with Nε-acetyl-L-lysine. Source data are provided as a Source Data file.

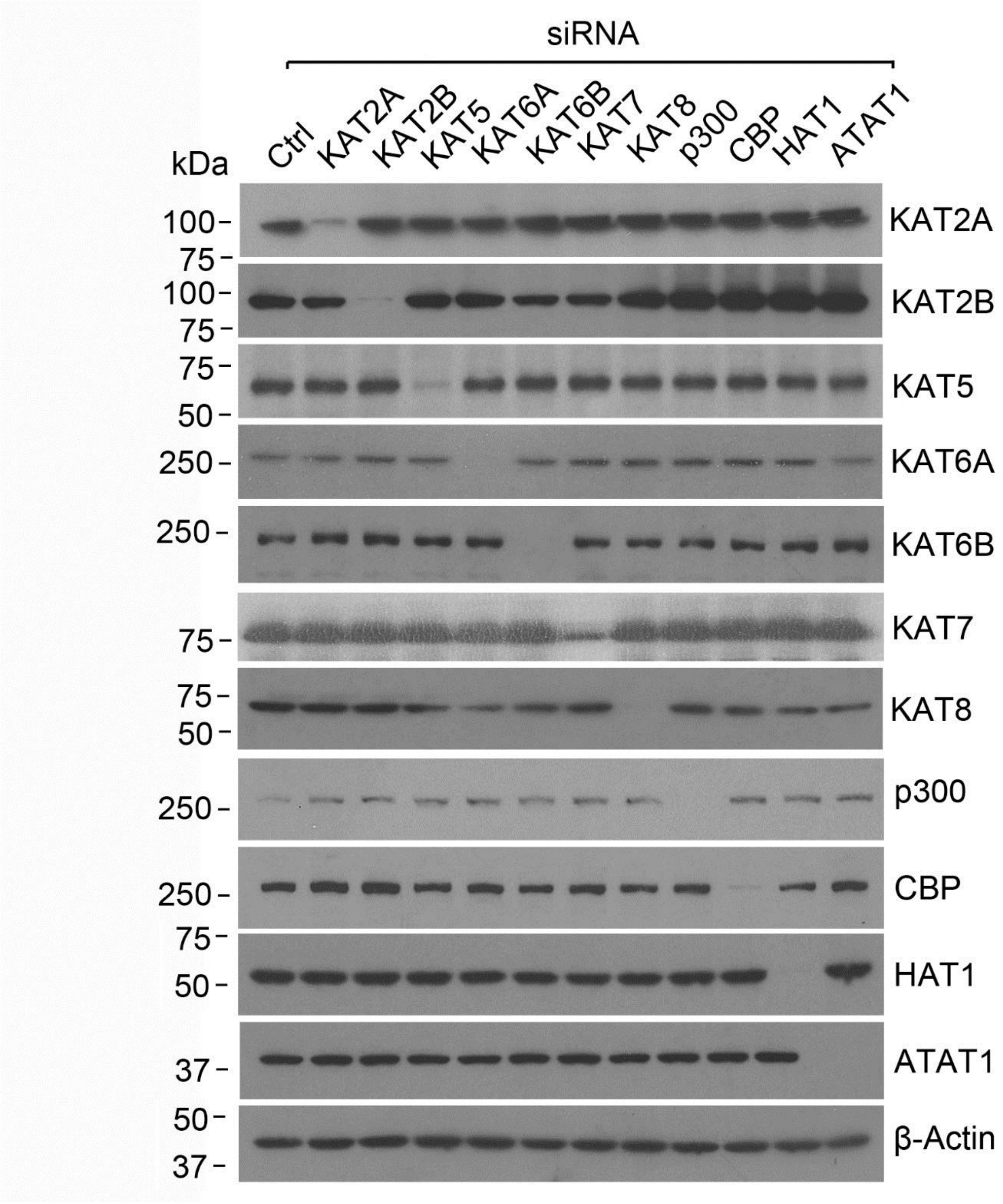

Supplementary Fig. 6 Knockdown of various acetyltransferase by an acetyltransferase siRNA library. Eleven acetyltransferase siRNAs were individually transfected into H1299 cells, followed by Western blot analysis of the expression of these acetyltransferases.
Source data are provided as a Source Data file.

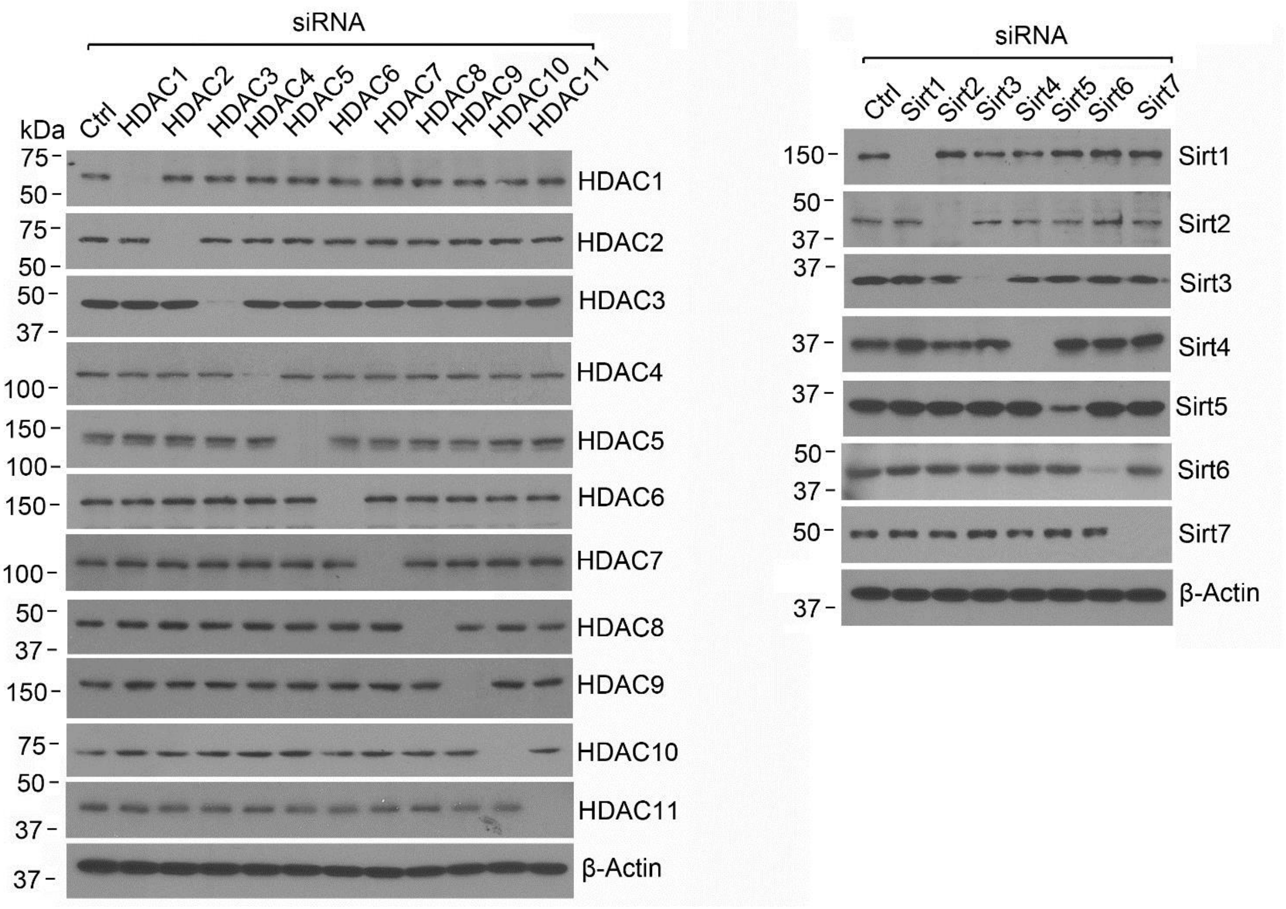

Supplementary Fig. 7 Knockdown of various protein deacetylases by a deacetylase siRNA library. Eighteen deacetylase siRNAs were individually transfected into H1299 cells, followed by Western blot analysis of the expression of these deacetylases. Source data are provided as a Source Data file.

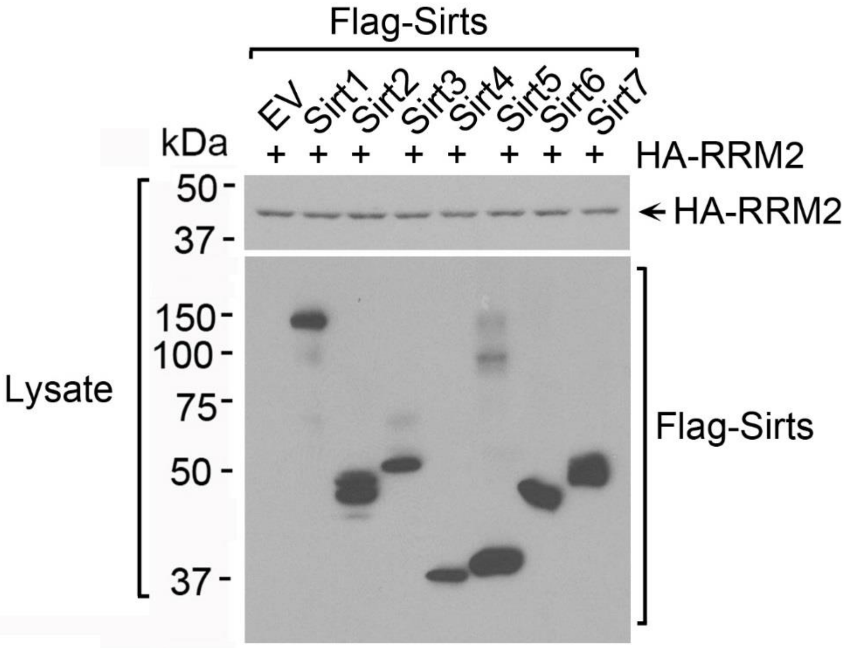

Supplementary Fig. 8 Expression of exogenous HA-RRM2 and various Flag-tagged Sirts. H1299 cells were co-transfected with HA-RRM2 along with empty vector (EV) or Flag-tagged Sirt1-7. Expression levels of HA-RRM2 and Flag-Sirt 1-7 in total cell lysates were analyzed by Western blot using HA or Flag antibody, respectively. Source data are provided as a Source Data file.

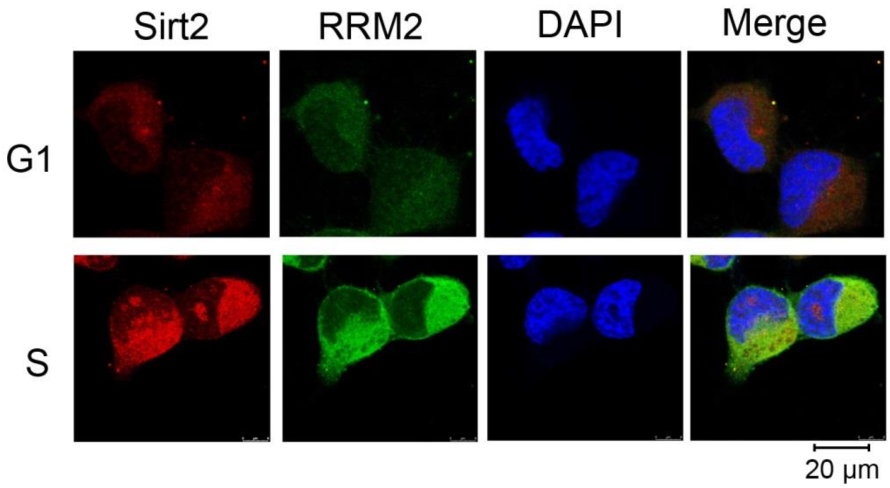

Supplementary Fig. 9 Co-localization of Sirt2 and RRM2 in G1 and S phase cells. S phase or G1 phase H1299 cells were collected at 2h or 14h time points following double thymidine block release. Co-immunostaining experiments were performed using RRM2 and Sirt2 antibodies in G1 and S cells. Scale bar: 20 μm.

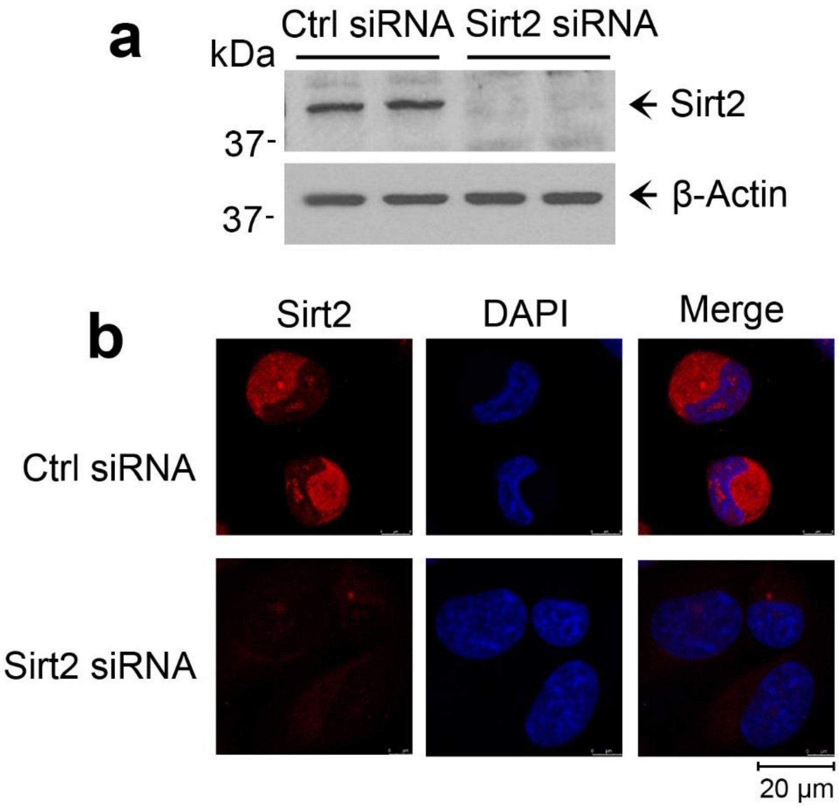

Supplementary Fig. 10 Knockdown of Sirt2 by siRNA. (**a**) Endogenous Sirt2 was knocked down in H1299 cells using Sirt2 siRNA. Sirt2 expression in H1299 and H1299 Sirt2 silenced cells was analyzed by Western blot using Sirt2 specific antibody. (**b**) Immunofluorescence staining experiments were performed using Sirt2 antibody in H1299 cells and H1299 Sirt2-silenced cells. Scale bar: 20 μm. Source data are provided as a Source Data file.

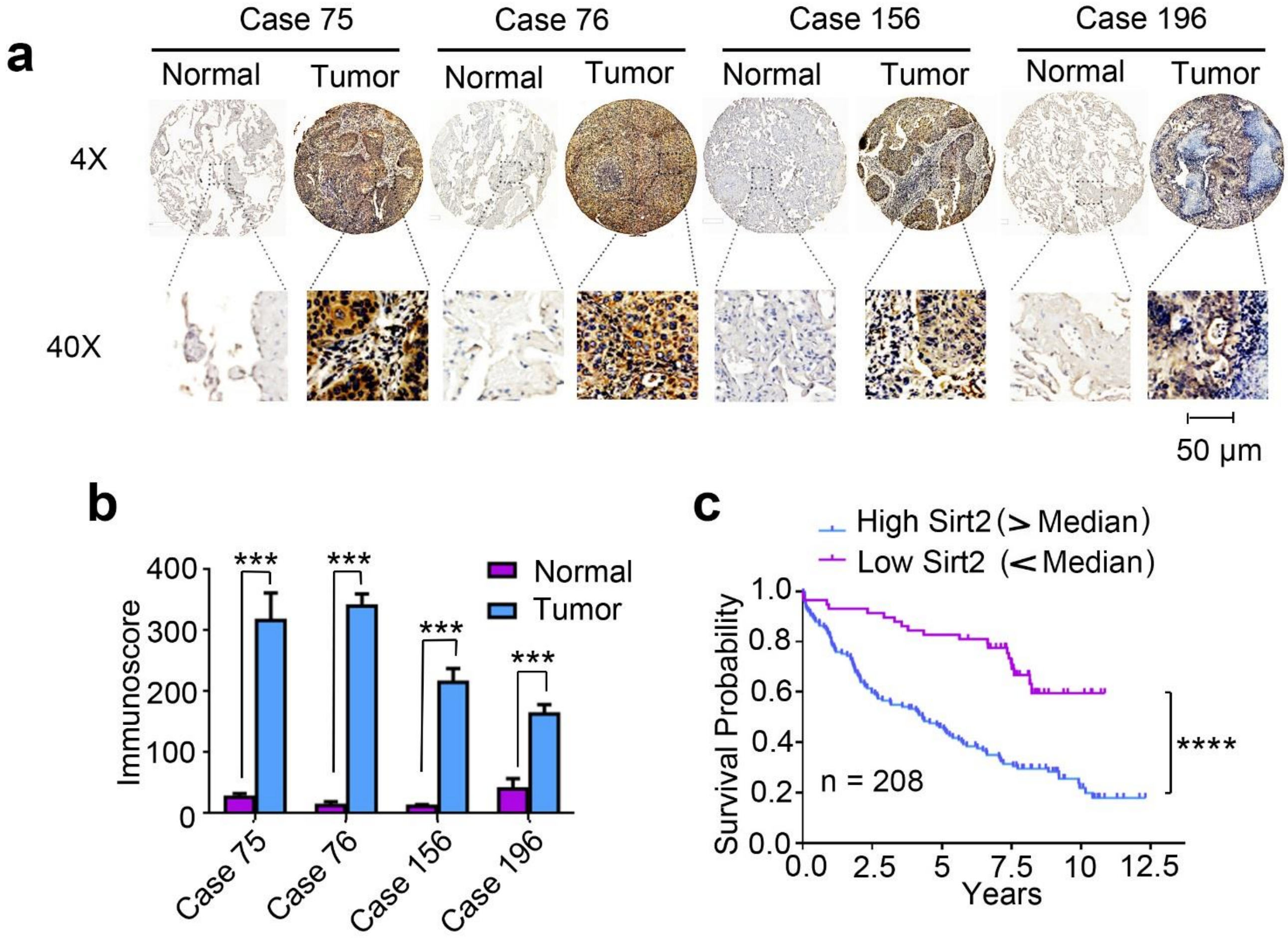

Supplementary Fig. 11 High levels of Sirt2 expression are associated with poor prognosis in NSCLC patients. (**a**) and (**b**) Sirt2 expression in normal lung tissues versus lung cancer tissues for representative cases was analyzed by IHC using Sirt2 antibody. Normal tissues are the adjacent normal lung tissues from the same cases. Sirt2 expression was quantified by immunoscore. The error bars indicate ± s.d. of three separate experiments. ***$P < 0.001$, by 2-tailed *t* test (**c**) Kaplan-Meier survival curve of NSCLC patients, n = 208. ****$P < 0.0001$, by log-rank test. Source data are provided as a Source Data file.

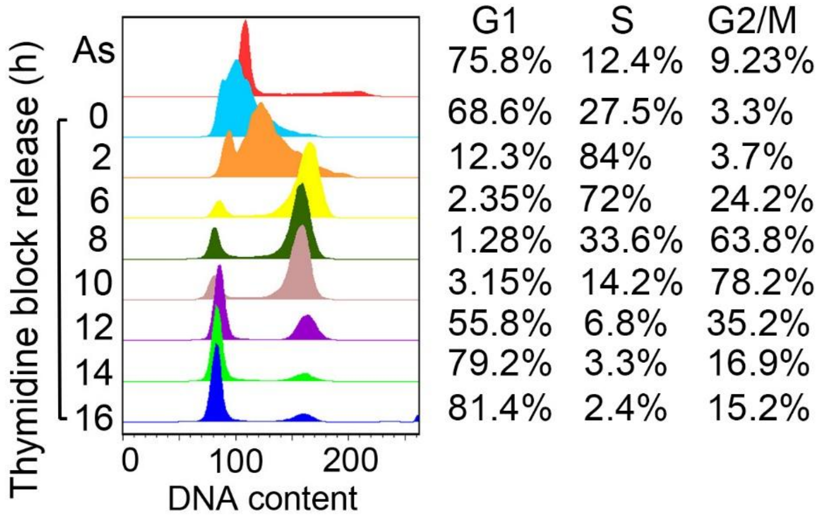

Supplementary Fig. 12 RRM2 acetylation at K95 occurs in a cell cycle dependent manner. H1299 cells were synchronized by double-thymidine block. After washing off thymidine, cells were released to normal medium for a time course up to 16h. Cell cycle was analyzed (left panel). Percentage of G1, S, and G2/M population was quantified.

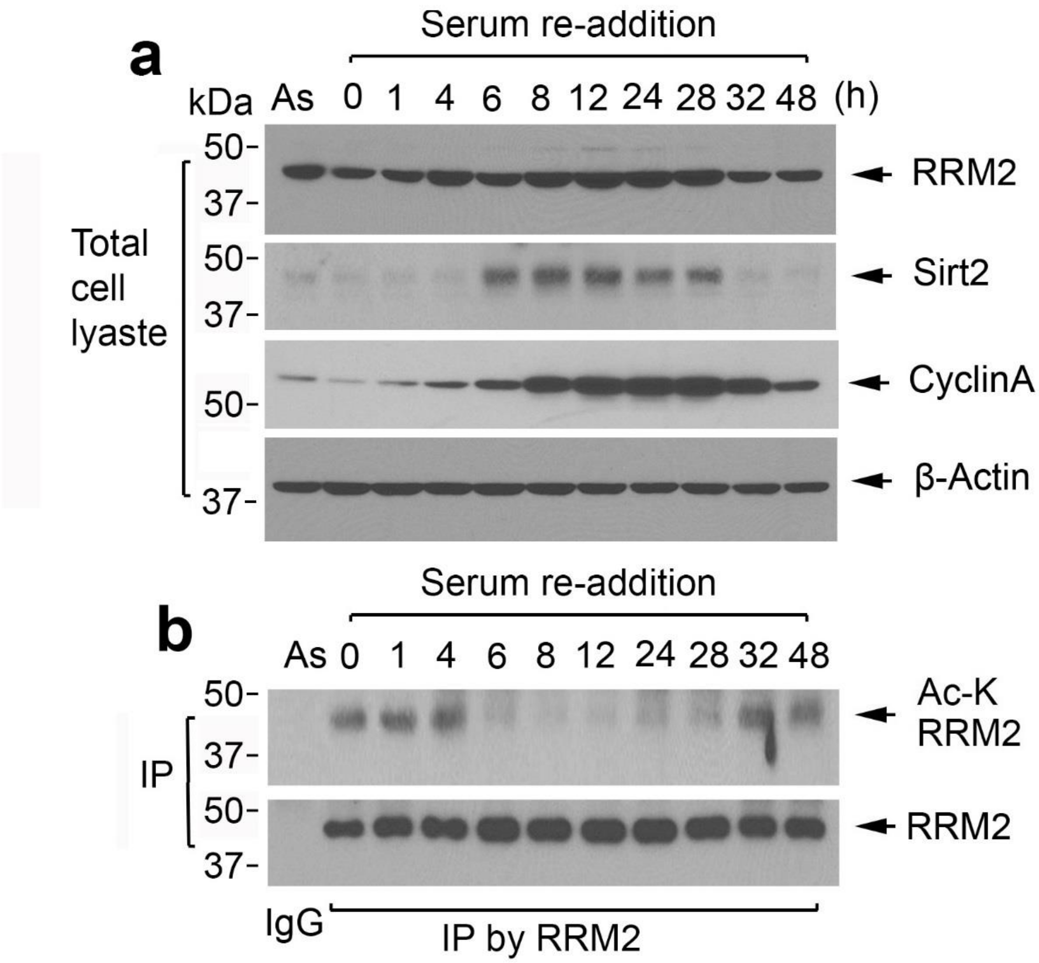

Supplementary Fig. 13 Effect of cell cycle progression on Sirt2 expression and RRM2 deacetylation. (**a**) H1299 cells were synchronized at the G0/G1 stage by serum starvation, followed by re-addition of serum (10% FBS) to allow cells to re-enter the cell cycle. Cells were collected at various time points. RRM2 and Sirt2 were analyzed by Western blot. Cyclin A was used as cell cycle S/G2 marker. (**b**) Samples from various time points were immunoprecipitated using RRM2 antibody. RRM2 acetylation was analyzed by Western blot with acetyl-specific antibody. Source data are provided as a Source Data file.

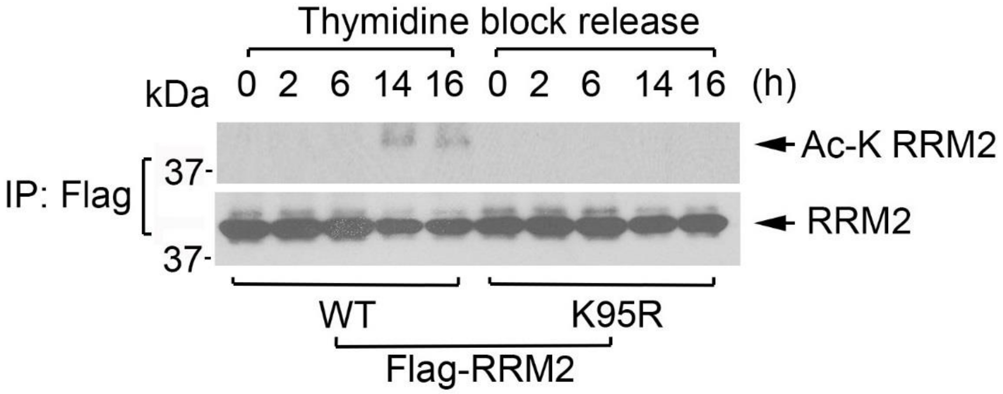

Supplementary Fig. 14 Acetylation of exogenous WT and K95R mutant RRM2 during cell cycle progression. H1299 cells were transfected with Flag-tagged WT or K95R mutant RRM2, followed by synchronization with double-thymidine block. After washing off thymidine, cells were released to normal medium for a time course up to 16h, followed by IP using Flag antibody. Acetylation of Flag-tagged RRM2 was analyzed by Western blot using acetyl-specific antibody. Source data are provided as a Source Data file.

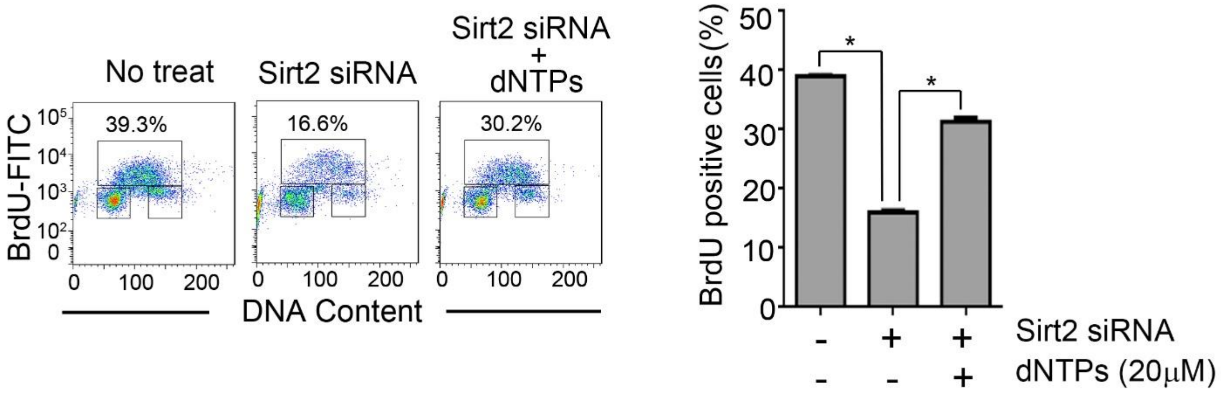

Supplementary Fig. 15 dNTPs reverses the inhibitory effect of *Sirt2* siRNA on DNA synthesis and/or cell proliferation. H1299 cells were transfected with Sirt2 siRNA, followed by BrdU incorporation and treatment with 20μM dNTPs for 24h. The percentage of BrdU positive cells were analyzed by FACS. The error bars indicate ± s.d. of three separate experiments. $*P < 0.05$, by 2-tailed *t* test. Source data are provided as a Source Data file.

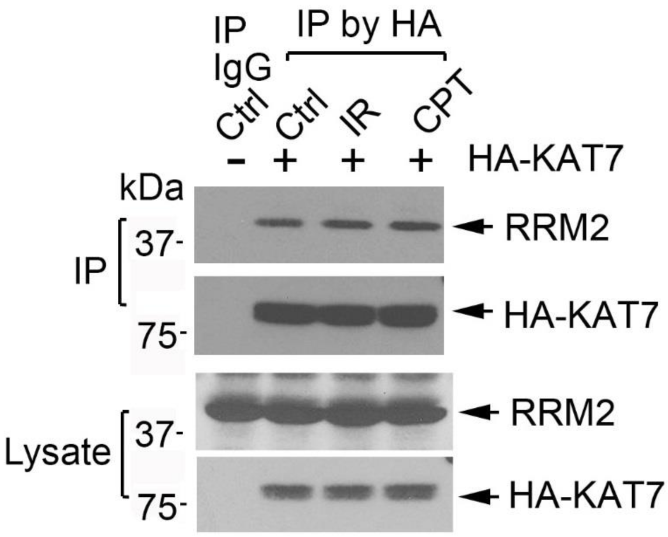

Supplementary Fig. 16 Effect of radiation and CPT-induced DNA damage on KAT7 and RRM2 association. H1299 cells were transfected with HA-tagged KAT7, followed by exposure to IR (10 Gy) or treatment with CPT (2 μM) for 2h. After lysis, co-IP experiments were performed using HA antibody, followed by Western blot using RRM2 antibody or HA antibody to detect RRM2 or HA-KAT7, respectively. Source data are provided as a Source Data file.

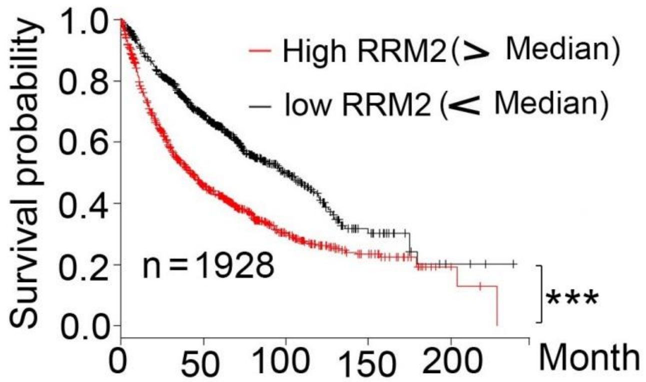

Supplementary Fig. 17 Increased RRM2 expression in tumor tissues is associated with poor prognosis of NSCLC patients. A correlation between overall survival of 1928 NSCLC patients and RRM2 expression was analyzed using Kaplan-Meier Plotter (www.kmplot.com), n = 1928. ***$P < 0.001$, by log-rank test.

Supplementary Methods

Materials

RRM1 (sc-11733, 1:500), RRM2 (sc-10846, 1:500), ATR (sc-1887, 1:500), Tip60 (sc-166323,1:1000), Sirt2 (sc-20966, 1:1000), KAT2A (sc-365321,1:500), KAT2B (sc-13124, 1:500), KAT5 (sc-166323,1:500), KAT8 (sc-81163, 1:500), p300 (sc-48343, 1:500), CBP (sc-7300, 1:500), HAT1 (sc-376268, 1:500), Sirt1(sc-135792, 1:1000), Sirt3 (sc-135796,1:500), Sirt4 (sc-135797, 1:500), Sirt5 (sc-271635, 1:500), Sirt6 (sc-517196, 1:500), Sirt7 (sc-365344, 1:500), HDAC1 (sc-81598, 1:500), HDAC2 (sc-55541, 1:500), HDAC3 (sc-81600, 1:500), HDAC4 (sc-56686, 1:500), HDAC5 (sc-133106,1:500), HDAC6 (sc-28386,1:500), HDAC7 (sc-74563, 1:500), HDAC8 (sc-365620, 1:500), HDAC9 (sc-398003, 1:500), HDAC10 (sc-365270, 1:500), HDAC11 (sc-390737, 1:500), Flag (sc-807, 1:500), HA (sc-57592, 1:1000) and β-actin (sc-58673, 1:2000) antibodies for Western blot, and RRM2 antibody (sc-10846) for IP (1:50) and for immunofluorescence staining (1:100), and Sirt2 (sc-28298) for IHC (1:100) and for immunofluorescence staining (1:100) were purchased from Santa Cruz (Santa Cruz, CA). Acetylated-lysine (#9441, 1:1000), cyclin A (#4656, 1:1000), pS/TQ ATM/ATR substrate (#9607, 1:1000) antibodies for Western blot, and PTMScan® Acetyl-Lysine Motif (Ac-K) Immunoaffinity Beads (13416) for pull-down were purchased from Cell Signaling Technology (Danvers, MA). KAT6A (ab41718, 1:1000), KAT6B (ab191994, 1:1000) and KAT7 (ab70183, 1:1000) antibodies for Western blot, Flag (sc-807, 1:100) for IP and Ki67 antibody (ab15580, 1:200) for IHC staining were purchased from Abcam (Cambridge, UK). ATR antibody (A300-138A, 1:100) for IP was obtained from Bethyl Laboratories (Montgomery, TX). Anti-Flag M2 affinity beads, Flag peptide, acetyl-CoA and N_{ε}-Acetyl-L-lysine were obtained from Sigma (St. Louis, MO). Trichostatin A (TSA) was purchased from Santa Cruz (Dallas, TX). Camptothecin (CPT), nicotinamide (NAM), and HA antibody (H6908, 1:100) for IP were purchased from Sigma-Aldrich (St. Louis, MO). Alexa Fluor 488 goat antimouse (A32723, 1:4000) and Alexa Fluor 555 goat anti-rabbit (A32732, 1: 4000) for immunofluorescence staining were purchased from Invitrogen (Carlsbad, CA). KU55933, VE821, and NU7441 were obtained from Selleckchem (Houston, TX). The pcDNA3.1-Flag-RRM2 plasmid was obtained from Dr. Michele Pagano (New York University School of Medicine). Flag-RRM1 and HA-KAT7 plasmids were purchased from Genescript (Nanjing, China). Flag-tagged Sirt1, Sirt2, Sirt3, Sirt4, Sirt5, Sirt6, Sirt7, and Sirt2 H181Y plasmids were obtained from Dr. David Yu (Emory University). pBABE-hygro was purchased from Addgene (Cambridge, MA). The RNAi libraries targeting 11 human acetyltransferases and 18 human deacetylases were purchased from Dharmacon (Lafayette, CO, USA). The dNTPs were purchased from Thermo Scientific (Waltham, MA). The pRSF-Duet1/MbtRNA$_{CUA}$/AcKRS-3 construct was obtained from Dr. Michael Lammers's laboratory (University of Cologne, Germany)[1, 2]. All reagents used were obtained from commercial sources unless otherwise stated.

Ribonucleotide reductase activity assay

Cellular RNR activity was assessed as we described previously[3]. Briefly, cells were lysed in a low salt homogenization buffer (10 mM Hepes pH 7.2, 2 mM DTT) by 30 passages through 27G syringe needle. After removal of cell debris by centrifugation at 16,000 g at 4°C for 20 min, the supernatants were passed through a Sephadex G25 spin column and the cell lysates were collected for the following assay. To measure RNR activity of recombinant RRM2 proteins,

293T cells were transiently transfected with Flag-RRM2 WT or mutant plasmids and the recombinant proteins were purified using anti-flag M2 beads. Then, the purified Flag-RRM2 and GST-RRM1 proteins were mixed at a 1:1 ratio (each 0.5 μg) for the following activity assay. The reaction mixture contained 50 mM Hepes buffer pH 7.2,10 mM DTT, 4 mM AMP-PNP, 20 μM $FeCl_3$, 2 mM magnesium acetate, 2mM ATP, 50 μM CDP and 100 μM C^{14}-CDP mixed with 600 μg cell lysates or purified proteins in a volume of 50 μl. After incubation at 37 ℃ for 1 h, 4 μl of 10 M perchloric acid was added for 15 min on ice. After centrifugation, the supernatant was transferred to a new tube and boiled for 20 min. 4 μl of a marker solution (60 mM CMP, 60 mM dCMP, and 60 mM dUMP plus 12 μl 5 M KOH) was added and then incubated on ice for 15 min. Samples were centrifuged at 14,000 rpm for 5 min. The resulting supernatant containing nucleotides was spotted on a TLC plate and separated by thin-layer chromatography. TLC plates were analyzed with quantification using the variable scanner Typhoon 9210 (GE health). RNR activity was calculated as ^{14}C-dCDP/ (^{14}C -CDP+^{14}C-dCDP).

Cell lysate, immunoprecipitation and Western blot

Cells were suspended in EBC buffer (0.5% NP-40, 50 mM Tris-HCl pH 7.6, 1 mM EDTA, and 1 mM β-mercaptoethanol) supplemented with protease inhibitor cocktail and deacetylase (Santa Cruz, CA), lysed by sonication, followed by centrifugation at 14,000×g for 10 min. The resulting supernatant was collected as the total cell lysate. Protein expression was analyzed by Western blot. For immunoprecipitation (IP)**,** the supernatant was incubated with agarose-conjugated antibody overnight at 4°C. After washing with EBC buffer 3 times and 1×PBS 2 times, beads were boiled in 30 μl of SDS-PAGE loading buffer, subjected to SDS-PAGE, and analyzed by Western blot. All uncropped and unprocessed scans of all blots were provided as a Source Data file.

Ac-K protein depletion by Ac-K immunoaffinity beads

Quantification of endogenous acetylated proteins was performed as described previously [4]. Cells were treated with 2 μM TSA and 10 mM NAM overnight, then lysed in EBC buffer (0.5% NP-40, 50 mM Tris-HCl pH 7.6, 1 mM EDTA,1 mM β-mercaptoethanol, 1 μM TSA, 5 mM NAM and protease inhibitor cocktail) with sonication. After centrifugation at 14,000×g for 10 min, cell lysates were incubated with PTMScan® Acetyl-Lysine Motif (Ac-K) Immunoaffinity Beads at 4 ^{0}C with rotation overnight, followed by centrifugation at 1000 × g for 3 min. Thus, acetylated and unacetylated proteins, including RRM2, were separated by beads and supernatants. Unacetylated RRM2 in supernatant and total RRM2 in total lysate before Ac-K depletion were analyzed by Western blot using RRM2 antibody, followed by quantification of Western bands using ImageJ software. The percentage of Ac-K RRM2 was calculated using formula: % Ac-K RRM2 = (Total RRM2 - Unacetylated RRM2)/Total RRM2 ×100.

Silencing of endogenous RRM2, KAT7, and Sirt2

RRM2, KAT7, and Sirt2 shRNA in pGIPZ lentiviral vector were purchased from Dharmacon (Thermo Fisher Scientific, CO). To restore expression of exogenous RRM2 in RRM2 silenced cells, 3'-UTR targeted RRM2 shRNA were chosen. The targeting sequences were: 5'-TAA ATC AAG CTA TTA TCG C-3' (shRNA-1); 5'-TAA AGG ACT GTT TAA TCC C-3' (shRNA-2). Sirt2 targeting sequences were: 5'-ACA GGA GAA GAA ACG CG CT-3' (shRNA1); 5'-TCC AAG GTC AGC TCG TCC A-3' (shRNA2). KAT7 targeting sequences were: 5'-TTC CTT ATA

TCG AAG CTG C-3' (shRNA1); 5'-TCC ATC TGA ACT ACT GCC T-3' (shRNA2). For lentivirus production, shRNA was co-transfected into 293FT cells with a lentivirus packaging plasmid mixture (pCMV-dR8.2 dvpr and pCMV-VSV-G) (System Biosciences, CA) using the NanoJuice transfection kit (EMD Chemical, Inc.). After 72 h, virus-containing medium supernatant was harvested by centrifugation at 20,000 × g. H1299 cells were infected with virus containing media in the presence of polybrene (8 µg/ml) and selected with 1 µg/ml puromycin. Since the RRM2 shRNA is directed to the 3'-UTR, the effect of the shRNA can be rescued by ectopically expressing WT or mutant RRM2. Re-expression of exogenous RRM2 was achieved with pBABE retrovirus vector. pBABE-hygro plasmids encoding RRM2 variants were co-transfected with packaging plasmids pCL-Eco and pCMV-VSV-G into 293T cells to generate retrovirus particles. H1299 cells with RRM2 knocked down were then infected with retrovirus harboring WT or RRM2 mutant(s). Stable clones were selected by hygromycin (500 µg/ml).

Colony formation assay
1×10^3 H1299 parental cells or RRM2 knock down cells re-expressing the indicated shRNA resistant mutants were seeded into 6-well plates. Cells were cultured in complete medium for 7-10 days and colonies were stained with 0.1% crystal violet in methanol, counted and normalized to parental cells.

Analysis of ATR-induced RRM2 phosphorylation *in vitro*
ATR kinase assay was performed as previously described[5]. To obtain active ATR kinase, H1299 cells were irradiated with 10 Gy. After 30 min, ATR was immunoprecipitated with ATR antibody. Immuno-complexes were incubated with purified RRM2 (1 µg) in 40 µl kinase buffer (10 mM HEPES pH 7.4, 50 mM NaCl, 10 mM $MgCl_2$,10 mM $MnCl_2$,1 mM DTT) containing protease inhibitor cocktail, 10 µM cold ATP, 25 µCi [γ-^{32}P] ATP at 30 °C for 1h. Reactions were terminated by boiling in SDS sample buffer, subjected to SDS-PAGE gel and analyzed by Typhoon 9210 phosphorimager (GE Healthcare).

BrdU labeling for analysis of cell cycle and cell proliferation
BrdU labeling was performed using a FITC BrdU flow kit (BD Pharmingen, San Diego, CA) according to the manufacturer's instruction. Briefly, cells were labeled with 10 µM BrdU for 30 min in culture medium and fixed with BD cytofix/cytoperm buffer provided in the kit. After DNA digestion, cells were stained with FITC-labeled anti-BrdU antibody for 20 min at room temperature in the dark, followed by the addition of 7-aminoactinomycin D (7-AAD) for staining of DNA content. Cell cycle distribution was determined by flow cytometry analysis of DNA content as described[6]. For dNTP replenishment, dNTPs were added into cells through hypotonic shift in KHB buffer (10 mM Hepes pH7.4, 30 mM KCl) for 24h as described previously[7, 8]. Cell proliferation was analyzed by calculating BrdU positive cells using flow cytometry as previously described [9].

Cell cycle analysis by PI staining
Cells were harvested and washed once with ice-cold 1×PBS. Cells were then fixed in cold 70% ethanol overnight at 4°C. Cells were washed twice with PBS and re-suspended in 1×PBS, followed by addition of RNAse (100 µg/mL) and incubation at room temperature for 60 min. Propidium iodide (50µg/ml) was added to cells and incubated at room temperature for 30 min. Cell cycle was analyzed by flow cytometry as described [10].

Cell synchronization
Cells were synchronized at the G1/S boundary by double thymidine block as described[11]. Briefly, dividing cells were incubated with 2mM thymidine for 16 h. After washing, cells were released into normal medium for 9h, followed by the addition of 2mM thymidine for another 16h. After double-thymidine block, cells were released to fresh media and analyzed at various time points.

Site-directed mutagenesis
The pcDNA3.1-Flag-RRM2 was used as a template for creating various RRM2 mutants by QuikChange site-directed mutagenesis kit (Agilent Technologies, Santa Clara, CA) according to the manufacturer's instructions. The primers used for mutagenesis were: RRM2 K30R, forward: 5'- AAG GGG CTC AGC TTG GTC GAC AGG GAG AAC ACG CCG CCG GCC CTG-3', reverse: 5'-CAG GGC CGG CGG CGT GTT CTC CCT GTC GAC CAA GCT GAG CCC CTT-3'; RRM2 K30Q, forward: 5'-AAG GGG CTC AGC TTG GTC GAC CAG GAG AAC ACG CCG CCG GCC CTG-3', reverse: 5'-CAG GGC CGG CGG CGT GTT CTC CTG GTC GAC CAA GCT GAG CCC CTT-3'; RRM2 K61R, forward: 5'-GAG CCC ACG GAG CCG AAA ACT CGA GCA GCT GCC CCC GGC GTG GAG-3', reverse: 5'-CTC CAC GCC GGG GGC AGC TGC TCG AGT TTT CGG CTC CGT GGG CTC-3'; RRM2 K61Q, forward: 5'-GAG CCC ACG GAG CCG AAA ACT CAA GCA GCT GCC CCC GGC GTG GAG-3', reverse: 5'-CTC CAC GCC GGG GGC AGC TGC TTG AGT TTT CGG CTC CGT GGG CTC-3'; RRM2 K95R, forward: 5'-CAT GAT ATC TGG CAG ATG TAT AGG AAG GCA GAG GCT TCC TTT TGG-3', reverse: 5'-CCA AAA GGA AGC CTC TGC CTT CCT ATA CAT CTG CCA GAT ATC ATG-3'; RRM2 K95Q, forward: 5'-CAT GAT ATC TGG CAG ATG TAT CAG AAG GCA GAG GCT TCC TTT TGG-3', reverse: 5'-CCA AAA GGA AGC CTC TGC CTT CTG ATA CAT CTG CCA GAT ATC ATG-3'; RRM2 K283R, forward: 5'-ATG TTC AAA CAC CTG GTA CAC AGA CCA TCG GAG GAG AGA GTA AGA-3', reverse: 5'-TCT TAC TCT CTC CTC CGA TGG TCT GTG TAC CAG GTG TTT GAA CAT-3'; RRM2 K283Q, forward: 5'-ATG TTC AAA CAC CTG GTA CAC CAA CCA TCG GAG GAG AGA GTA AGA-3',reverse: 5'-TCT TAC TCT CTC CTC CGA TGG TTG GTG TAC CAG GTG TTT GAA CAT-3'. After 26 PCR cycles, the amplification product was digested with Dpn I to remove the non-mutated WT template and transformed into DH5α for amplification. The resulting RRM2 mutants in pcDNA3.1 were confirmed by sequencing.

Expression and purification of recombinant RRM1 and RRM2 proteins
RRM2 WT and 95Q proteins were cloned into pET20b (+) vector containing His_6 tag between HindIII and XhoI sites. *E. coli* Rosetta (DE3) were transformed and grown at 37°C in LB (Luria-Bertani) media with shaking. When the density reached an OD600 of 0.6, protein expression was induced with IPTG (0.2 mM) at 18 °C for 18h. Bacteria were then harvested and resuspended in 40 ml buffer A (20 mM Tris, pH 8.0, 120 mM NaCl and 10% glycerol) per 1 L of bacteria and lysed by sonication. The lysate was centrifuged at 14,000 rpm for 30 min, and the supernatant was incubated with HisPur Cobalt Resin (Thermo Scientific) at 4 °C for 2h. After washing with buffer A, the his_6-RRM2 proteins were eluted with buffer B (20 mM Tris, pH 8.0, 120 mM NaCl, 300 mM imidazole and 10% glycerol). The eluted protein was dialyzed with buffer A and stored at -80°C. For preparation of GST-RRM1 protein, the RRM1 cDNA was cloned into pGEX-4T1 between SalI and NotI sites. Transformed *E. coli* Rosetta (DE3) (EMD Millipore) harboring pGEX-4T1-RRM1 plasmids were grown in LB broth at 37° C with shaking

at 250 rpm and induced with 0.1 mM IPTG upon OD600 reaching 0.6. Bacteria were then incubated at 18°C with shaking for 18h, followed by lysis in buffer A with sonication. After centrifugation, supernatant was applied to a glutathione sepharose column (GE healthcare) and washed with 10 column volumes of buffer G. GST-RRM1 proteins were eluted with buffer C (20 mM Tris-HCl, pH 8.0, 10 mM glutathione) and stored at -80° C for experimental use.

In vitro acetylation assay

WT or K95R Flag tagged RRM2 proteins were purified with anti-Flag M2 affinity beads (Sigma) from H1299 cells. Full length recombinant KAT7 protein was purchased from Active Motif (#31489, Carlsbad, CA, USA). The protein acetylation assay was performed as previously described[12]. In the standard assay, a 50 µl reaction mixture contained 2 µg of Flag-RRM2, 300 ng of KAT7, 5 µg of acetyl-CoA in acetylation assay buffer (50 mM HEPES, pH8.0, 10% glycerol,0.1mM EDTA, 1mM dithiothreitol, 1 mM phenylmethylsulfony fluoride). The reaction mixture was incubated at 30°C for 4h, followed by SDS–PAGE and Western blot using acetyl-specific antibody.

Electron paramagnetic resonance spectroscopy

EPR measurements were performed as described previously[13]. Briefly, purified RRM2 protein was adjusted to 3.0 µg/µl in buffer A (50 mM Tris-HCl pH=7.4, 100 mM NaCl, 10% v/v glycerol), and Fe^{2+} was added from stock solution (final Fe^{2+}/RRM2 ratio, 3.0). Samples were loaded into 4 mm outer diameter EPR tubes. After 10 min at 295 K, samples were frozen in liquid nitrogen-chilled isopentane (140 K), and stored in liquid nitrogen. Continuous-wave (CW)-EPR measurements were performed. EPR spectra were collected on a Bruker Elexsys E500 spectrometer by using a Super High Q Cavity (Bruker ER 4122SHQE) and Bruker ER4131VT system with nitrogen-flow cooling for temperature control. The following experimental conditions were used: microwave frequency, 9.4504 GHz; modulation frequency, 100 kHz; modulation amplitude, 0.4 mT; microwave power, 20.5 mW; temperature, 97 K; number of scans averaged, 32. Sample spectra were corrected by subtraction of a buffer baseline spectrum (average of 64 scans).

DNA fiber assay

DNA fiber spreads were performed as described previously[3]. Briefly, cells were first labeled with 5’chlorodeoxyuridine (CldU, 200 µM) for 20 min. After incubation with the first label and washing, cells were then labeled with iododeoxyuridine (IdU, 100 µM) for another 20 min. After labeling, 2 µl of cell suspension ($1×10^6$ cells/ml) in cold PBS were spotted onto a microscope slide and mixed with 12 µl lysis buffer (0.5% SDS, 50 mM EDTA, 200 mM Tris-Cl) for 10 min at room temperature. Then, slides were tilted to 15° to allow the fibers to spread along the slide. Fibers were then fixed with methanol and acetic acid (3:1), followed by denaturation in 2.5 M HCl for 80 min. After washing with 1×PBS, slides were blocked with 10% goat serum for at least 1 h, and then incubated with primary antibodies against IdU (mouse anti-BrdU clone B44) and CldU (rat anti-BrdU BU1/75(ICR1)) and secondary antibodies (Alexa Fluor 488 (green) goat anti-mouse and Alexa Fluor (red) 555 goat anti-rat) for 1 h. After mounting, images were collected with a Zeiss Axioplan2 microscope (Axioplan, Zeiss) and analyzed by Zeiss AxioVision software. The DNA length was calculated as 2.596 kb/µm and the fork rate (kb/min) was calculated from the length of fluorescent signal (kb) divided by the time of the pulse.

Protein cross-linking analysis
RRM2 proteins at 0.25 mg/ml in 20 mM sodium phosphate buffer (pH 8.0) were cross-linked with 2 mM disuccinimidyl suberate (DSS) for 30 min at room temperature. The reaction was stopped with 50 mM Tris-HCl, pH 7.5 and analyzed by SDS-PAGE.

Immunofluorescence
Cells were grown on chamber slides (BD Falcon, MA), washed with cold 1□□PBS, fixed with 4.0% paraformaldehyde in 1×PBS for 10 min at room temperature, and permeabilized with 0.5% Triton X-100 in 1×PBS for 15 min. After blocking with 10% normal goat serum (Life technologies, Carlsbad, CA) for 1h, samples were incubated with the indicated primary antibody at 4℃ overnight. After washing, samples were incubated with 1:4000 dilution of Alexa Fluor 488 goat anti-mouse IgG (green) or Alexa Fluor 555 goat anti-rabbit IgG (red) for 45 min at room temperature in the dark. After washing, samples were mounted with Prolong Gold antifade reagent containing DAPI (Invitrogen). Images were captured using LSM 510 confocal microscope (Zeiss, Sweden).

Establishment of lung cancer xenografts in mice
Lung cancer xenografts were generated as we described previously[14]. All animal experiments were approved by the Institutional Animal Care and Use Committee of Emory University. Forty 6-week-old male nu/nu nude mice were purchased from Harlan (IN, US) and housed under pathogen-free conditions. These forty mice were randomly divided into 8 groups without any selective criteria. Each group has 5 mice (n=5). 1×10^{7} H1299 parental or RRM2 silenced cells expressing exogenous WT or various acetyl-mimetic RRM2 mutants were subcutaneously inoculated into mouse flanks. Tumor growth was monitored by measuring tumor volume for 4 weeks using a caliper and calculated with the formula: $V = (L\times W^2)/2$ (L, length; W, width) as described[14].

Supplementary References

1. de Boor S*, et al.* Small GTP-binding protein Ran is regulated by posttranslational lysine acetylation. *Proc Natl Acad Sci U S A* **112**, E3679-3688 (2015).

2. Knyphausen P*, et al.* Insights into Lysine Deacetylation of Natively Folded Substrate Proteins by Sirtuins. *J Biol Chem* **291**, 14677-14694 (2016).

3. Xie M*, et al.* Bcl2 induces DNA replication stress by inhibiting ribonucleotide reductase. *Cancer Res* **74**, 212-223 (2014).

4. Li M, Luo J, Brooks CL, Gu W. Acetylation of p53 inhibits its ubiquitination by Mdm2. *J Biol Chem* **277**, 50607-50611 (2002).

5. Tibbetts RS*, et al.* A role for ATR in the DNA damage-induced phosphorylation of p53. *Genes & development* **13**, 152-157 (1999).

6. Pfister SX*, et al.* Inhibiting WEE1 Selectively Kills Histone H3K36me3-Deficient Cancers by dNTP Starvation. *Cancer cell* **28**, 557-568 (2015).

7. Malinsky J, Koberna K, Stanek D, Masata M, Votruba I, Raska I. The supply of exogenous deoxyribonucleotides accelerates the speed of the replication fork in early S-phase. *Journal of cell science* **114**, 747-750 (2001).

8. Koberna K*, et al.* Nuclear organization studied with the help of a hypotonic shift: its use permits hydrophilic molecules to enter into living cells. *Chromosoma* **108**, 325-335 (1999).

9. Placencio VR, Ichimura A, Miyata T, DeClerck YA. Small Molecule Inhibitors of Plasminogen Activator Inhibitor-1 Elicit Anti-Tumorigenic and Anti-Angiogenic Activity. *PloS one* **10**, e0133786 (2015).

10. Chen G*, et al.* Targeting Mcl-1 enhances DNA replication stress sensitivity to cancer therapy. *J Clin Invest* **128**, 500-516 (2018).

11. Chabes AL, Bjorklund S, Thelander L. S Phase-specific transcription of the mouse ribonucleotide reductase R2 gene requires both a proximal repressive E2F-binding site and an upstream promoter activating region. *The Journal of biological chemistry* **279**, 10796-10807 (2004).

12. Wang W, Pan K, Chen Y, Huang C, Zhang X. The acetylation of transcription factor HBP1 by p300/CBP enhances p16INK4A expression. *Nucleic acids research* **40**, 981-995 (2012).

13. Zhou B*, et al.* Structural basis on the dityrosyl-diiron radical cluster and the functional differences of human ribonucleotide reductase small subunits hp53R2 and hRRM2. *Molecular cancer therapeutics* **9**, 1669-1679 (2010).

14. Han B*, et al.* Small-Molecule Bcl2 BH4 Antagonist for Lung Cancer Therapy. *Cancer Cell* **27**, 852-863 (2015).
