# Supplementary material for: Acetylation regulates ribonucleotide reductase activity and cancer cell growth
Source: Nat Commun. 2019 Jul 19;10:3213. doi: 10.1038/s41467-019-11214-9 (PMC6642173; doi:10.1038/s41467-019-11214-9)
Supplement: Supplementary file 3 — Reporting Summary [file 41467_2019_11214_MOESM3_ESM.pdf]

nature research

Corresponding author(s): Xingming Deng

Last updated by author(s): Jun 10, 2019

Reporting Summary

Nature Research wishes to improve the reproducibility of the work that we publish. This form provides structure for consistency and transparency in reporting. For further information on Nature Research policies, see Authors & Referees and the Editorial Policy Checklist.

Statistics

For all statistical analyses, confirm that the following items are present in the figure legend, table legend, main text, or Methods section.

n/a	Confirmed	
☐	☒	The exact sample size (*n*) for each experimental group/condition, given as a discrete number and unit of measurement
☐	☒	A statement on whether measurements were taken from distinct samples or whether the same sample was measured repeatedly
☐	☒	The statistical test(s) used AND whether they are one- or two-sided<br>*Only common tests should be described solely by name; describe more complex techniques in the Methods section.*
☐	☒	A description of all covariates tested
☒	☐	A description of any assumptions or corrections, such as tests of normality and adjustment for multiple comparisons
☐	☒	A full description of the statistical parameters including central tendency (e.g. means) or other basic estimates (e.g. regression coefficient) AND variation (e.g. standard deviation) or associated estimates of uncertainty (e.g. confidence intervals)
☐	☒	For null hypothesis testing, the test statistic (e.g. *F*, *t*, *r*) with confidence intervals, effect sizes, degrees of freedom and *P* value noted<br>*Give P values as exact values whenever suitable.*
☒	☐	For Bayesian analysis, information on the choice of priors and Markov chain Monte Carlo settings
☒	☐	For hierarchical and complex designs, identification of the appropriate level for tests and full reporting of outcomes
☒	☐	Estimates of effect sizes (e.g. Cohen's *d*, Pearson's *r*), indicating how they were calculated

Our web collection on statistics for biologists contains articles on many of the points above.

Software and code

Policy information about availability of computer code

Data collection	No software was used
Data analysis	No software was used

For manuscripts utilizing custom algorithms or software that are central to the research but not yet described in published literature, software must be made available to editors/reviewers. We strongly encourage code deposition in a community repository (e.g. GitHub). See the Nature Research guidelines for submitting code & software for further information.

Data

Policy information about availability of data

All manuscripts must include a data availability statement. This statement should provide the following information, where applicable:

- Accession codes, unique identifiers, or web links for publicly available datasets
- A list of figures that have associated raw data
- A description of any restrictions on data availability

Data availability:
The data that support the findings of this study, its supplementary information and source data file are included in the paper, and are available from the corresponding author (X. Deng) upon reasonable request. The mass spectrometry proteomics source data are available via ProteomeXchange with identifier PXD014105.

Field-specific reporting

Please select the one below that is the best fit for your research. If you are not sure, read the appropriate sections before making your selection.

☒ Life sciences ☐ Behavioural & social sciences ☐ Ecological, evolutionary & environmental sciences

For a reference copy of the document with all sections, see nature.com/documents/nr-reporting-summary-flat.pdf

Life sciences study design

All studies must disclose on these points even when the disclosure is negative.

Sample size	Sample sizes were chosen to detect a minimum effect size of 1.5 with at least 80% power and a type I error of 0.05 for each comparison.
Data exclusions	No samples or animals have been excluded from the analysis.
Replication	The measures taken for experiments were repeated at least three times, and data are presented as mean plus/minus standard deviation (s.d) of three separate experiments. All attempts at replication were successful. All findings can be replicated.
Randomization	Forty mice were randomly divided into 8 groups without any selective criteria.
Blinding	Blinding was not relevant to the study because methods for group allocation, data collection and all related analyses were designed in advance.

Reporting for specific materials, systems and methods

We require information from authors about some types of materials, experimental systems and methods used in many studies. Here, indicate whether each material, system or method listed is relevant to your study. If you are not sure if a list item applies to your research, read the appropriate section before selecting a response.

Materials & experimental systems

n/a	Involved in the study	
☐	☒	Antibodies
☐	☒	Eukaryotic cell lines
☒	☐	Palaeontology
☐	☒	Animals and other organisms
☒	☐	Human research participants
☒	☐	Clinical data

Methods

n/a	Involved in the study	
☐	☐	ChIP-seq
☐	☒	Flow cytometry
☐	☐	MRI-based neuroimaging

Antibodies

Antibodies used	Please see details in "Materials" section in "Supplementary Methods". RRM1 (sc-11733, 1:500), RRM2 (sc-10846, 1:500), ATR (sc-1887, 1:500), Tip60 (sc-166323,1:1000), Sirt2 (sc-20966, 1:1000), KAT2A (sc-365321,1:500), KAT2B (sc-13124, 1:500), KAT5 (sc-166323,1:500), KAT8 (sc-81163, 1:500), p300 (sc-48343, 1:500), CBP (sc-7300, 1:500), HAT1 (sc-376268, 1:500), Sirt1(sc-135792, 1:1000), Sirt3 (sc-135796,1:500), Sirt4 (sc-135797, 1:500), Sirt5 (sc-271635, 1:500), Sirt6 (sc-517196, 1:500), Sirt7 (sc-365344, 1:500), HDAC1 (sc-81598, 1:500), HDAC2 (sc-55541, 1:500), HDAC3 (sc-81600, 1:500), HDAC4 (sc-56686, 1:500), HDAC5 (sc-133106,1:500), HDAC6 (sc-28386,1:500), HDAC7 (sc-74563, 1:500), HDAC8 (sc-365620, 1:500), HDAC9 (sc-398003, 1:500), HDAC10 (sc-365270, 1:500), HDAC11 (sc-390737, 1:500), Flag (sc-807, 1:500), HA (sc-57592, 1:1000) and β-actin (sc-58673, 1:2000) antibodies for Western blot, and RRM2 antibody (sc-10846) for IP (1:50) and for immunofluorescence staining (1:100), and Sirt2 (sc-28298) for IHC (1:100) and for immunofluorescence staining (1:100) were purchased from Santa Cruz (Santa Cruz, CA). Acetylated-lysine (#9441, 1:1000), cyclin A (#4656, 1:1000), pS/TQ ATM/ATR substrate (#9607, 1:1000) antibodies for Western blot, and PTMScan® Acetyl-Lysine Motif (Ac-K) Immunoaffinity Beads (13416) for pull-down were purchased from Cell Signaling Technology (Danvers, MA). KAT6A (ab41718, 1:1000), KAT6B (ab191994, 1:1000) and KAT7 (ab70183, 1:1000) antibodies for Western blot, Flag (sc-807, 1:100) for IP and Ki67 antibody (ab15580, 1:200) for IHC staining were purchased from Abcam (Cambridge, UK). ATR antibody (A300-138A, 1:100) for IP was obtained from Bethyl Laboratories (Montgomery, TX). Anti-Flag M2 affinity beads, Flag peptide, acetyl-CoA and Nε-Acetyl-L-lysine were obtained from Sigma (St. Louis, MO). Trichostatin A (TSA) was purchased from Santa Cruz (Dallas, TX). Camptothecin (CPT), nicotinamide (NAM), and HA antibody (H6908, 1:100) for IP were purchased from Sigma-Aldrich (St. Louis, MO). Alexa Fluor 488 goat antimouse (A32723, 1:4000) and Alexa Fluor 555 goat anti-rabbit (A32732, 1: 4000) for immunofluorescence staining were purchased from Invitrogen (Carlsbad, CA).
Validation	According to company source of each antibody name and catalog number as above, and we found the data sheet on the manufacturer's website and validated relevant citations and antibody profiles, and clearly indicated in the manuscript.

Eukaryotic cell lines

Policy information about cell lines

Cell line source(s)	H460, H1299, BEAS-2B and HBEC3 cell lines were obtained from the American Type Culture Collection.
Authentication	No further authentication for these cell lines was carried out by authors.
Mycoplasma contamination	These cell lines were tested for mycoplasma and had no mycoplasma contamination.
Commonly misidentified lines (See ICLAC register)	Misidentified lines were not used.

Animals and other organisms

Policy information about studies involving animals; ARRIVE guidelines recommended for reporting animal research

Laboratory animals	6-week-old male nu/nu nude mice were used in this study.
Wild animals	Wild animals were not used.
Field-collected samples	No field-collected samples in this study
Ethics oversight	All animal experiments were approved by the Institutional Animal Care and Use Committee (IACUC) of Emory University.

Note that full information on the approval of the study protocol must also be provided in the manuscript.

ChIP-seq

Data deposition

☐ Confirm that both raw and final processed data have been deposited in a public database such as GEO.

☐ Confirm that you have deposited or provided access to graph files (e.g. BED files) for the called peaks.

Data access links *May remain private before publication.*	No ChIP-seq in this study
Files in database submission	No ChIP-seq in this study
Genome browser session (e.g. UCSC)	No ChIP-seq in this study

Methodology

Replicates	No ChIP-seq in this study
Sequencing depth	No ChIP-seq in this study
Antibodies	No ChIP-seq in this study
Peak calling parameters	No ChIP-seq in this study
Data quality	No ChIP-seq in this study
Software	No ChIP-seq in this study

Flow Cytometry

Plots

Confirm that:

☒ The axis labels state the marker and fluorochrome used (e.g. CD4-FITC).

☒ The axis scales are clearly visible. Include numbers along axes only for bottom left plot of group (a 'group' is an analysis of identical markers).

☒ All plots are contour plots with outliers or pseudocolor plots.

☒ A numerical value for number of cells or percentage (with statistics) is provided.

Methodology

Sample preparation	Human lung cancer cell line samples were directly collected from culture dishes.
Instrument	FACSCanto™ II Flow Cytomter 643554, BD Biosciences
Software	Flowjo 7.6.1
Cell population abundance	No sorting procedures were used in the analyses.
Gating strategy	Cells were gated based on the clearly separated BrdU positive population, compared with un-stained control cells.

☐ Tick this box to confirm that a figure exemplifying the gating strategy is provided in the Supplementary Information.

Magnetic resonance imaging

Experimental design

Design type	No magnetic resonance imaging was used in this study
Design specifications	No magnetic resonance imaging was used in this study
Behavioral performance measures	No magnetic resonance imaging was used in this study

Acquisition

Imaging type(s)	No magnetic resonance imaging was used in this study
Field strength	No magnetic resonance imaging was used in this study
Sequence & imaging parameters	No magnetic resonance imaging was used in this study
Area of acquisition	No magnetic resonance imaging was used in this study

Diffusion MRI ☐ Used ☒ Not used

Preprocessing

Preprocessing software	No magnetic resonance imaging was used in this study
Normalization	No magnetic resonance imaging was used in this study
Normalization template	No magnetic resonance imaging was used in this study
Noise and artifact removal	No magnetic resonance imaging was used in this study
Volume censoring	No magnetic resonance imaging was used in this study

Statistical modeling & inference

Model type and settings	No magnetic resonance imaging was used in this study
Effect(s) tested	No magnetic resonance imaging was used in this study

Specify type of analysis: ☐ Whole brain ☐ ROI-based ☐ Both

Statistic type for inference (See Eklund et al. 2016)	No magnetic resonance imaging was used in this study
Correction	No magnetic resonance imaging was used in this study

Models & analysis

n/a	Involved in the study
☒	☐ Functional and/or effective connectivity
☒	☐ Graph analysis
☒	☐ Multivariate modeling or predictive analysis
